# Influence of Chelator Type on the Efficiency and Mechanisms of Electrokinetic Remediation of Copper- and Lead-Contaminated Loess

**DOI:** 10.3390/toxics14080658

**Published:** 2026-07-26

**Authors:** Yunxiao Jin, Longping Luo, Shixu Zhang, Zheng Yuan

**Affiliations:** 1 School of Intelligent Construction and Civil Engineering, Luoyang Institute of Science and Technology, Luoyang 471023, China; jinyunxiao@lit.edu.cn; 2 School of Civil Engineering, Xi’an University of Architecture and Technology, Xi’an 710055, China; 3 School of Civil Engineering and Architecture, Xi’an University of Technology, Xi’an 710048, China

**Keywords:** electrokinetic remediation, loess, copper- and lead-contamination, chelating agent, removal efficiency

## Abstract

With the continued advancement of industrialization and urbanization in northwestern China, heavy metal contamination of loess sites has become an increasingly serious environmental issue. Heavy metals such as copper (Cu) and lead (Pb) are toxic, persistent, and readily retained by the mineral-pore framework of loess, which makes remediation difficult and threatens regional ecological security and human health. This study investigated electrokinetic (EK) remediation of artificially prepared loess co-contaminated with Cu and Pb using tartaric acid (TA), citric acid (CA), and disodium ethylenediaminetetraacetate (EDTA) as catholytes. Cu was generally removed more effectively than Pb because Pb was less adsorbed and immobilized more strongly. All three chelators enhanced metal desorption and migration through complexation, particularly in the cathode-side section. EDTA produced the greatest enhancement because it formed stable, negatively charged complexes with Cu and Pb over a broad pH range. Relative to the deionized-water control, EDTA increased the overall removal efficiencies of Cu and Pb to 55.4% and 27.2%, respectively, and promoted their transfer from the soil to the anolyte. These findings demonstrate that chelator-assisted EK treatment, particularly with EDTA, can effectively improve Cu and Pb removal from loess, while field application requires control of energy use, residual chelator, and post-treatment metal mobility.

## 1. Introduction

According to the National Soil Pollution Survey Bulletin released in 2014, 16.1% of soil sampling sites in China exceeded the corresponding pollution standards, among which inorganic pollutants accounted for 82.8%, with heavy metals such as copper (Cu) and lead (Pb) being the dominant contaminants [1]. In recent years, the continuous adjustment of China’s industrial layout and regional development strategy has accelerated the transfer of industries to northwestern (NW) China. While this has promoted sectional economic growth, it has also increased soil environmental risks in this region [2]. Under the influence of mining, metal smelting, equipment manufacturing, industrial emissions, and waste disposal, heavy metals such as Cu and Pb have been continuously introduced into soils, leading to local accumulation and exceedance of environmental standards [3]. Loess is widely distributed in NW China and is characterized by a well-developed pore structure, relatively high carbonate content, weak alkalinity, and strong structural sensitivity. Once contaminated by heavy metals, pollutants are prone to adsorption, retention, and accumulation in the loess matrix, defined here as the mineral-pore framework formed by silt- and clay-sized particles, carbonates, organic matter, and pore water, which makes both contamination assessment and remediation more difficult [4]. In addition, heavy metal pollution in soil is concealed, cumulative, and persistent, and can pose long-term threats to ecological security and human health through plant uptake and food-chain transfer [4]. Therefore, the efficient remediation of Cu- and Pb-contaminated loess is of great environmental and engineering significance.

Various techniques have been developed for the remediation of heavy metal-contaminated soils, including soil replacement [5], solidification/stabilization [6,7], chemical leaching [8], and phytoremediation [9]. However, these methods often suffer from limited applicability, long treatment periods, possible secondary pollution, or strong dependence on contaminant type [10]. In contrast, electrokinetic (EK) remediation has attracted increasing attention because of its relatively high efficiency, low environmental disturbance, good applicability to low-permeability soils, and suitability for both inorganic and some organic contaminants [11]. EK remediation relies on the application of a direct-current electric field across the soil, which drives contaminants toward the electrodes through electromigration, electroosmosis, and electrophoresis, thereby achieving separation or removal [12]. For heavy metal-contaminated soils, the EK removal efficiency largely depends on whether the contaminants can exist as mobile ionic species or charged complexes. Nevertheless, practical EK treatment requires a direct-current power supply, durable electrodes, electrolyte circulation and pH-control units. Energy consumption, reagent use, electrode maintenance, wastewater handling, and scale-dependent soil heterogeneity can increase costs and constrain field implementation [13].

However, the intrinsic properties of loess can significantly restrict EK performance. Loess in NW China usually contains abundant carbonates, is weakly alkaline, and exhibits strong acid-buffering capacity. As a result, the H^+^ generated at the anode cannot effectively propagate toward the cathode during EK treatment, which limits heavy metal desorption from the soil. In addition, loess tends to collapse upon wetting, causing pore compression and permeability reduction, which further restrict the migration pathways of desorbed metals and reduce overall mass transfer efficiency. For Cu- and Pb-contaminated loess, Pb generally shows a stronger tendency toward adsorption and fixation than Cu, making its desorption and migration even more difficult. Consequently, Cu and Pb in loess are subject to multiple constraints, including difficult desorption, limited migration, and low removal efficiency, which is one of the main reasons for the restricted engineering application of EK in loess. Therefore, developing appropriate enhancement strategies for EK remediation of heavy metal-contaminated loess remains an urgent research need.

Current research on enhanced EK remediation of heavy metal-contaminated soils has mainly focused on two strategies: optimization of the EK process itself and its coupling with other remediation technologies. The first approach commonly includes pH regulation and the addition of enhancers such as chelating agents. pH control can be achieved through acid preconditioning, catholyte pH adjustment, or the use of ion-exchange membranes, thereby suppressing cathodic alkalization and promoting heavy metal desorption and migration [13,14,15]. Chelating agents can form soluble and charged complexes with metal ions, improving heavy metal stability and mobility over a relatively wide pH range. Previous studies have shown that chelating agents such as nitrilotriacetic acid (NTA), diethylenetriaminepentaacetic acid (DTPA), ethylene glycol tetraacetic acid (EGTA), ethylenediamine-N,N′-disuccinic acid (EDDS), citric acid, and EDTA can significantly enhance the electrokinetic removal of heavy metals from contaminated soils or sediments [16,17,18]. The second approach involves coupling EK with other technologies, such as phytoremediation, chemical leaching, and permeable reactive barriers. Among these, EK-permeable reactive barrier systems have been widely studied and have shown promising enhancement effects [19,20].

Although enhanced EK remediation has been extensively investigated, studies focusing specifically on Cu- and Pb-contaminated loess remain limited. Moreover, not all the enhancement strategies are suitable for loess. pH regulation is generally more effective in soils with relatively weak buffering capacity, such as sandy soils and kaolin, whereas the high carbonate content and strong buffering capacity of loess often limit the effectiveness of acidification alone. Coupled technologies such as permeable reactive barriers usually require contaminants to already possess sufficient desorption and migration ability, which is often not the case in loess because of the combined effects of adsorption/fixation and structural constraints. In comparison, chelating agents can form soluble and charged complexes with most heavy metal ions over a broad pH range, thereby simultaneously improving both desorption and migration [21]. From this perspective, chelator-enhanced EK appears to be a promising strategy for improving the remediation efficiency of Cu- and Pb-contaminated loess.

Despite growing interest in enhanced EK remediation, the comparative performance of different chelators and their effects on Cu and Pb transport in strongly buffered loess remain insufficiently understood. Thus, the aim of this study was to comprehensively compare the effectiveness of 0.1 mol/L tartaric acid, citric acid, and EDTA in the EK remediation of Cu- and Pb-contaminated loess and to clarify the electrochemical and transport mechanisms responsible for the observed differences.

## 2. Materials and Methods

### 2.1. Experimental Materials

#### 2.1.1. Loess Used in the Experiments

Loess in NW China was mainly formed by the long-term deposition of aeolian dust and has subsequently undergone pedogenesis, leaching, and post-depositional modification, resulting in typical sectional depositional characteristics. The geomorphic units in the study area mainly include loess ridges, loess hills, and bedrock-incised gullies, indicating relatively complex topographic conditions. Borehole data show that the loess cover in this area mainly consists of an underlying paleosol layer and an overlying Malan loess layer (Q_3_), both of which rest on sandstone bedrock. The paleosol layer is approximately 22 to 31 m thick, whereas the Malan loess layer is approximately 2 to 5 m thick [22].

**Figure 1:**
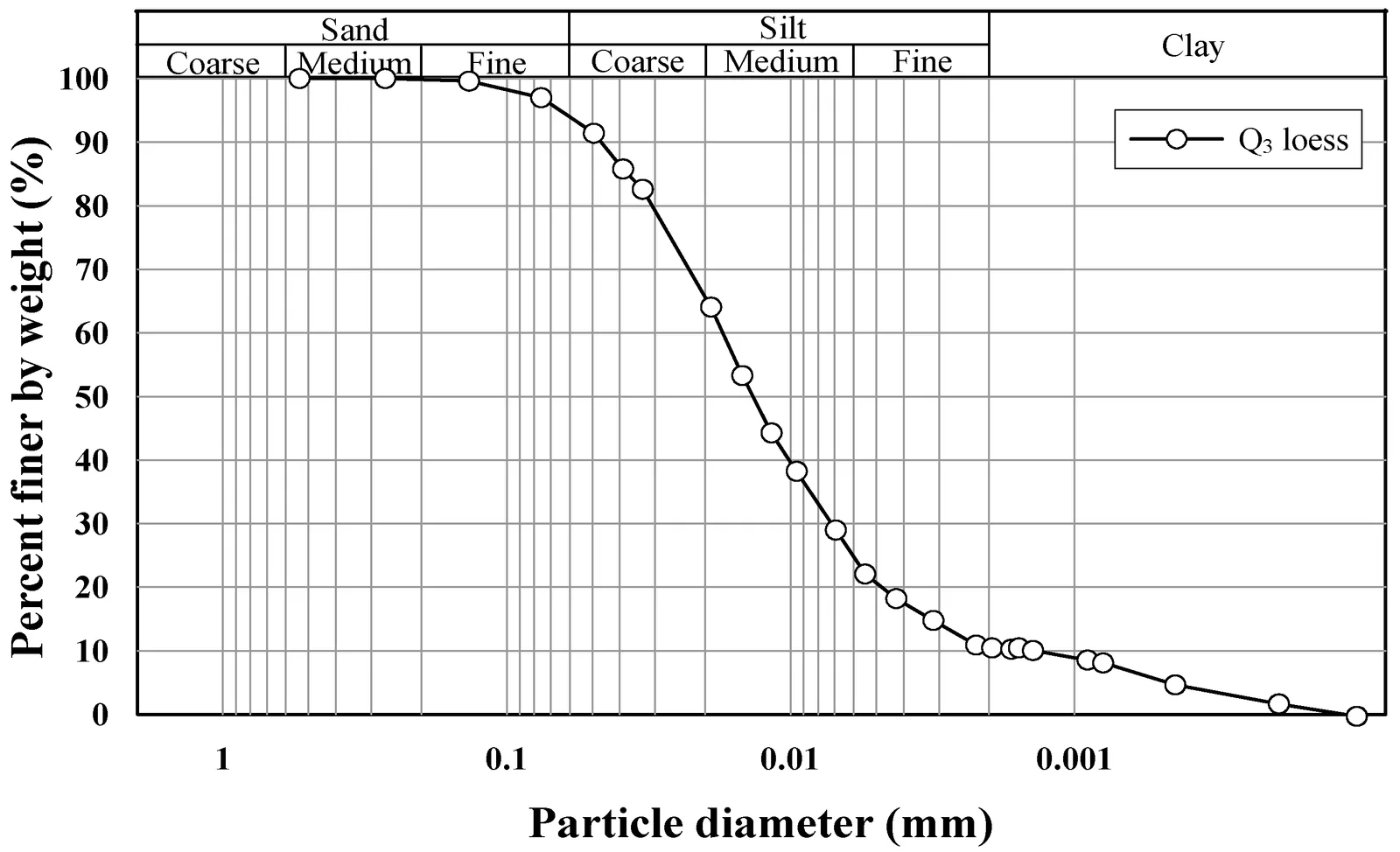
Particle-size distribution of the loess.

The loess used in this study was collected from Lantian County, Shaanxi Province, approximately 22 km southeast of Xi’an, at a depth of 3.0–4.5 m. The physicochemical properties listed in Table 1 were determined using standardized procedures. Natural water content, bulk unit weight, specific gravity, liquid limit, plastic limit, void ratio, and permeability were measured in accordance with GB/T 50123-2019 [23]. Particle-size distribution was measured using a WJL-602 laser particle-size analyzer (Jinan Winner Particle Instrument Stock Co., Ltd., Jinan, China). Soil pH and electrical conductivity were measured in soil–water suspensions using calibrated pH and conductivity meters; organic matter was determined by dichromate oxidation, and the BET specific surface area was measured by N2 adsorption. The soil consisted of 87.40% silt, 9.30% clay, and 3.30% sand (see Figure 1), and was classified as low-plasticity clay (CL).

In addition, the background Cu and Pb contents in the undisturbed loess were determined using the same analytical procedure described in Section 2.4. Briefly, the soil was oven-dried, digested with HNO_3_, and analyzed by atomic absorption spectrophotometry. Neither Cu nor Pb was detected above the corresponding method detection limits. Because the instrumental analysis was performed after acid digestion, the results represented not only dissolved or free ionic Cu and Pb, but also exchangeable, adsorbed, precipitated, and other acid-extractable forms. Therefore, the tested loess was considered free of detectable background Cu and Pb contamination and was suitable as a model soil for the subsequent remediation experiments with artificially prepared Cu- and Pb-contaminated loess. Moreover, its typical physicochemical characteristics of silty loess in northwestern China make it representative for investigating the electrochemical migration and spatial distribution of Cu and Pb in loess pore media [23,24,25,26].

**Table 1:** Physicochemical properties of the loess [27,28,29].

Property	Loess
Sand (%)	3.3
Silt (%)	87.4
Clay (%)	9.3
Void ratio, e	0.898
Bulk unit weight, γ (kN·m^−3^)	16.2
Specific gravity, G_s_	2.69
Water content, ω_n_ (%)	16.5
Liquid limit, ω_L_ (%)	31.6
Plastic limit, ω_P_ (%)	19.5
USCS symbol	CL
Permeability (m·s^−1^)	2.55 × 10^−6^
Organic matter (mg·g^−1^)	4.1
pH	7.8
Electrical conductivity (μs·cm^−1^)	244
BET specific surface area (m^2^·g^−1^)	24.1
Composition of ions	
Ca^2+^ (mg·kg^−1^)	126
Mg^2+^ (mg·kg^−1^)	40
Na^+^ (mg·kg^−1^)	103
K^+^ (mg·kg^−1^)	4.6

#### 2.1.2. Chemical Reagents

Copper nitrate [Cu(NO_3_)_2_, analytical grade (AR), purity > 99.0%] and lead nitrate [Pb(NO_3_)_2_, AR, purity > 99.0%] were used as the sources of Cu and Pb contamination, respectively. Cu(NO_3_)_2_ was purchased from Shanghai Macklin Biochemical Co., Ltd. (Shanghai, China), and Pb(NO_3_)_2_ was purchased from Sinopharm Chemical Reagent Shaanxi Co., Ltd. (Xian, China). Tartaric acid (AR, purity > 99.0%) was purchased from Shanghai Adamas Reagent Co., Ltd. (Shanghai, China). Citric acid (AR, purity > 99.0%) and disodium ethylenediaminetetraacetate (EDTA-2Na, AR, purity > 99.0%) were purchased from Tianjin Damao Chemical Reagent Factory (Tianjin, China).

#### 2.1.3. Electrode Material

Electrokinetic geosynthetics (EKG) were used as the electrode material in this study. The electrodes were supplied by Henan Angzhi Engineering Consulting Co., Ltd. (Zhengzhou, China). The electrode dimensions were 150 mm × 150 mm × 8 mm. Its specific surface area, determined by nitrogen adsorption, was 8.900 m^2^/g. Compared with conventional metal electrodes, EKG provides better interfacial contact and more stable electrical conductivity, which is beneficial for sustaining ion transport and electrochemical reactions during electrokinetic remediation. Therefore, it was considered suitable for the EK remediation experiments on loess conducted in this study.

### 2.2. Sample Preparation

**Figure 2:**
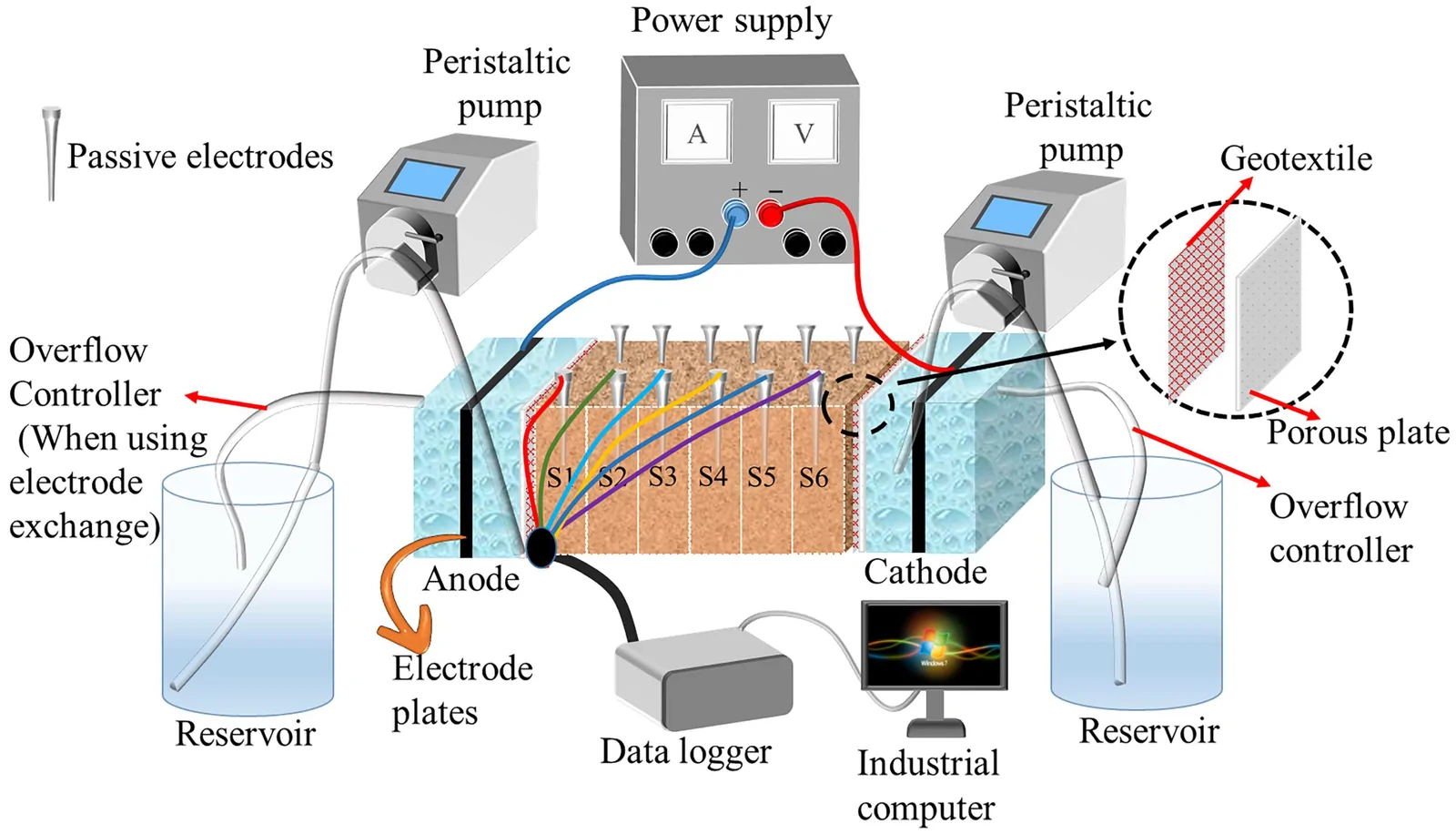
Schematic illustration of the electrokinetic reactor (Hu et al. (2022), Liu et al. (2025) and Wu et al. (2026) [27,28,29]). © 2022 Hu, Cheng, Wen and Kang. & © 2025 & © 2026 by the authors. Licensee MDPI, Basel, Switzerland. This article is an open access article distributed under the terms and conditions of the Creative Commons Attribution (CC BY) license (https://creativecommons.org/licenses/by/4.0/).

The collected loess was dried to an air-dry state at room temperature until its mass was constant, gently disaggregated, and passed through a 2 mm sieve. The target Cu and Pb concentrations were each set at 500 mg kg^−1^ to represent a clearly contaminated condition while remaining within the concentration range commonly used in laboratory EK studies, thereby allowing the differences among chelators to be compared under identical contamination loading. Based on the soil-compartment dimensions, sample height, target degree of compaction of 0.80, and water content of 15%, the required dry soil, deionized water, Cu(NO_3_)_2_, and Pb(NO_3_)_2_ were calculated. The salts were dissolved in deionized water and mixed thoroughly with the loess in accordance with GB/T 50123-2019 [23]. The prepared soil was sealed and equilibrated for 48 h. It was then placed into the reactor in three layers and compacted uniformly; each layer surface was scarified before the next layer was added to minimize interlayer discontinuities. Each EK condition was independently repeated three times, and the curves and spatial profiles present the mean values. Because the six sections within one reactor are spatially correlated subsamples rather than independent treatment replicates, section-to-section differences in Figure 2 are interpreted descriptively, and no section-wise significance claim is made.

### 2.3. Electrokinetic Reactor and Experimental Design

EK remediation systems reported in the literature generally consist of a soil chamber and two electrode compartments located at both ends of the reactor, and this configuration has been widely applied to the treatment of heavy metal- and organic-contaminated soils [24]. In most previous laboratory studies, however, the soil chamber volume was relatively small. For example, Yuan et al. (2016) [24] used a soil chamber of 150 mm × 38 mm × 60 mm for black soil contaminated with heavy metals, Liu et al. (2016) [25] employed a reactor with a chamber size of 100 mm × 80 mm for copper-contaminated tailings, and Millán et al. (2020) [26] adopted a chamber of 14 cm × 100 mm × 80 mm for herbicide-contaminated soils. Although these small-scale systems are useful for investigating the basic principles of EK remediation, several studies have shown that larger soil volumes can provide more representative process parameters and are more valuable for scaling up to practical applications. In particular, larger-scale reactors are more conducive to capturing spatial variations in current, electroosmotic flow, and other process responses, thereby improving the reliability of experimental results for engineering interpretation. Based on these considerations, a large-scale electrokinetic reactor was designed in this study.

As shown in Figure 2, the reactor consisted of a plexiglass soil chamber, two electrode compartments, a pair of electrodes, two electrolyte reservoirs, two peristaltic pumps, a DC-regulated power supply, and a data acquisition system. The internal dimensions of the soil chamber were 500 mm × 150 mm × 150 mm, while each electrode compartment measured 100 mm × 150 mm × 150 mm. The two electrode compartments were connected to the ends of the soil chamber and separated from the soil by geotextile and porous plates. To facilitate the analysis of contaminant transport and spatial distribution, the soil chamber was divided into six sections, denoted as S1–S6. An overflow controller was used to maintain a constant electrolyte level in the electrode compartments.

A pair of electrodes was installed in the two electrode compartments to generate the electric field. Two electrolyte reservoirs, each with a size of 150 mm in diameter and 250 mm in height, were connected to the corresponding electrode compartments. When pH adjustment was required, 1.0 mol/L NaOH or 1.0 mol/L HCl was added in small aliquots to the catholyte reservoir while the electrolyte was continuously circulated. During the experiments, electric current, pH, electrical conductivity (EC), and cumulative electroosmotic flow (EOF) were monitored using the data acquisition system. At the end of each EK test, the removal efficiencies of Cu and Pb were determined and compared under different experimental conditions.

As summarized in Table 2, 0.1 mol/L EKTA, EKCA, and EKEDTA solutions were used as catholytes, while deionized water (EKDW) served as the control catholyte. All the other operating conditions were kept constant.

### 2.4. Analytical Procedure

After EK treatment, the samples were oven-dried and subsequently digested with HNO_3_. The resulting digestion solutions were analyzed using an atomic absorption spectrophotometer (Hitachi High-Tech Corporation, Tokyo, Japan). The removal efficiency of heavy metals during EK treatment was calculated according to the following equation [30,31]:

Removal efficiency(%) = [(C_0_ − C_f_)/C_0_] × 100% (1)

where C_0_ is the initial heavy metal concentration (mg/kg) of loess, and C_f_ is the final concentration of heavy metals (mg/kg) after EK treatment in loess.

## 3. Results and Discussion

### 3.1. Variation in pH

**Figure 3:**
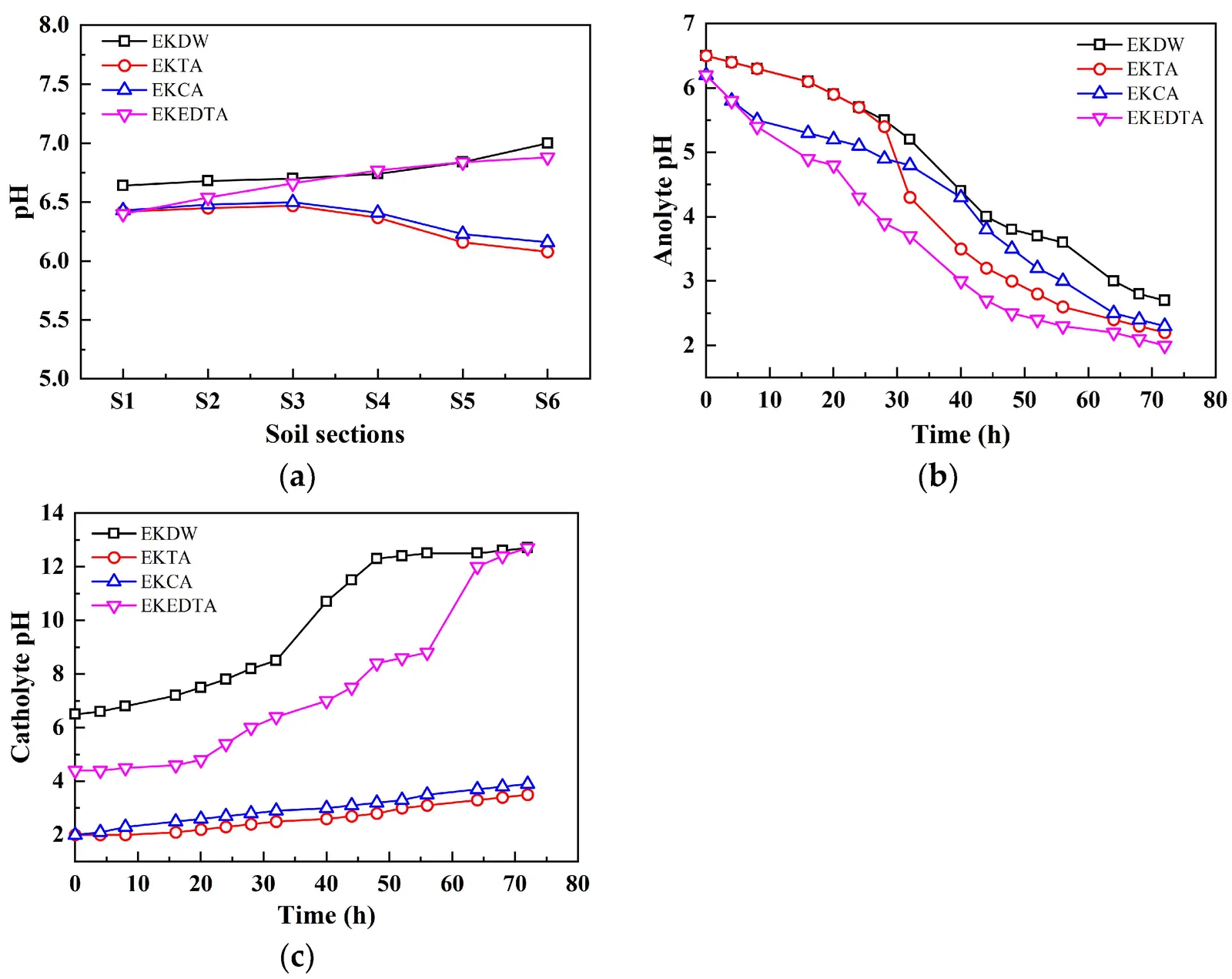
Variations in pH of the soil profiles and the electrolytes in the electrode compartments after electrokinetic remediation: (**a**) pH distribution in different soil sections; (**b**) variation in anolyte pH; (**c**) variation in catholyte pH.

Figure 3 shows the spatial distribution of soil pH at the end of the EK remediation tests, together with the temporal variations in the pH of the anolyte and catholyte. As shown in Figure 3, the soil pH profiles in EKDW and EKEDTA were generally similar, both exhibiting a gradual increase from the anode side to the cathode side. In contrast, the soil pH in EKTA and EKCA first increased and then decreased along the reactor length. Meanwhile, with increasing remediation time, the anolyte pH continuously decreased, whereas the catholyte pH gradually increased.

Under the applied direct-current electric field, water electrolysis occurred at both electrodes, and the corresponding reactions are given as follows [27]:

(2) $$\mathrm{Anode}:\;2H_{2}O-4e^{-}\to O_{2}↑+4H^{+}\;\;\;E_{0}=-1.229\;V$$

Anode: 2H_2_O − 4e^−^ → O_2_↑ + 4H^+^   E_0_ = −1.229 V (3)

(4) $$\mathrm{Cathode}:\;2H_{2}O+2e^{-}\to H_{2}↑+2OH^{-}\;\;\;E_{0}=-0.828\;V$$

Cathode: 2H_2_O + 4e^−^ → H_2_↑ + 2OH^−^   E_0_ = −0.828 V (5)

where E_0_ is the standard electrode potential.

According to these reactions, H^+^ was continuously generated at the anode, whereas OH^−^ was continuously produced at the cathode. Consequently, the anolyte became progressively acidic, while the catholyte gradually became alkaline. Because the migration rate of H^+^ toward the cathode is approximately 1.75 times that of OH^−^ toward the anode [32], a distinct acid-alkaline zonation gradually developed in the soil. As a result, the soil pH decreased near the anode and increased near the cathode.

The pH distribution in the soil was mainly governed by the electrolyte pH and the intrinsic acid-buffering capacity of the loess. For EKDW and EKEDTA, the anolyte pH in both tests gradually decreased to 2–3, whereas the catholyte pH increased to approximately 12. Accordingly, the soil pH in both groups generally increased from S1 to S6. However, compared with EKDW, EKEDTA exhibited a higher current during the experiment, which led to greater H^+^ generation at the anode and therefore stronger acidification in the anodic section, resulting in a lower pH near the anode. In addition, EKEDTA used weakly acidic EDTA-2Na as the catholyte, which provided a certain buffering effect against the OH^−^ generated at the cathode. This partially inhibited the diffusion and migration of OH^−^ into the soil, and therefore the soil pH in the cathode-side section (S6) of EKEDTA was lower than that of EKDW. For EKTA and EKCA, although tartaric acid and citric acid have similar acidity, both are more acidic than EDTA-2Na and thus exhibit stronger neutralization and buffering effects toward OH^−^. As a result, the catholyte in EKTA and EKCA remained acidic throughout the experiment, causing the soil near the cathode to be exposed to an acidic environment from the initial stage of remediation. Under such conditions, the original acid-buffering capacity of the soil in the cathodic section was continuously and rapidly consumed. By comparison, in the early stage of the tests, the current in the anodic section was relatively low, resulting in limited H^+^ production and slower consumption of the soil acid-buffering capacity near the anode. Consequently, both EKTA and EKCA eventually exhibited lower soil pH on the cathode side than on the anode side. In addition, the current in EKCA was higher than that in EKTA during the later stage of the experiment, resulting in a faster generation of OH^−^ near the cathode and a greater consumption of the buffering capacity of citric acid. Therefore, the ability of citric acid to suppress cathodic alkalization weakened in the later stage, causing the soil pH near the cathode in EKCA to be slightly higher than that in EKTA.

### 3.2. Variation in EC

**Figure 4:**
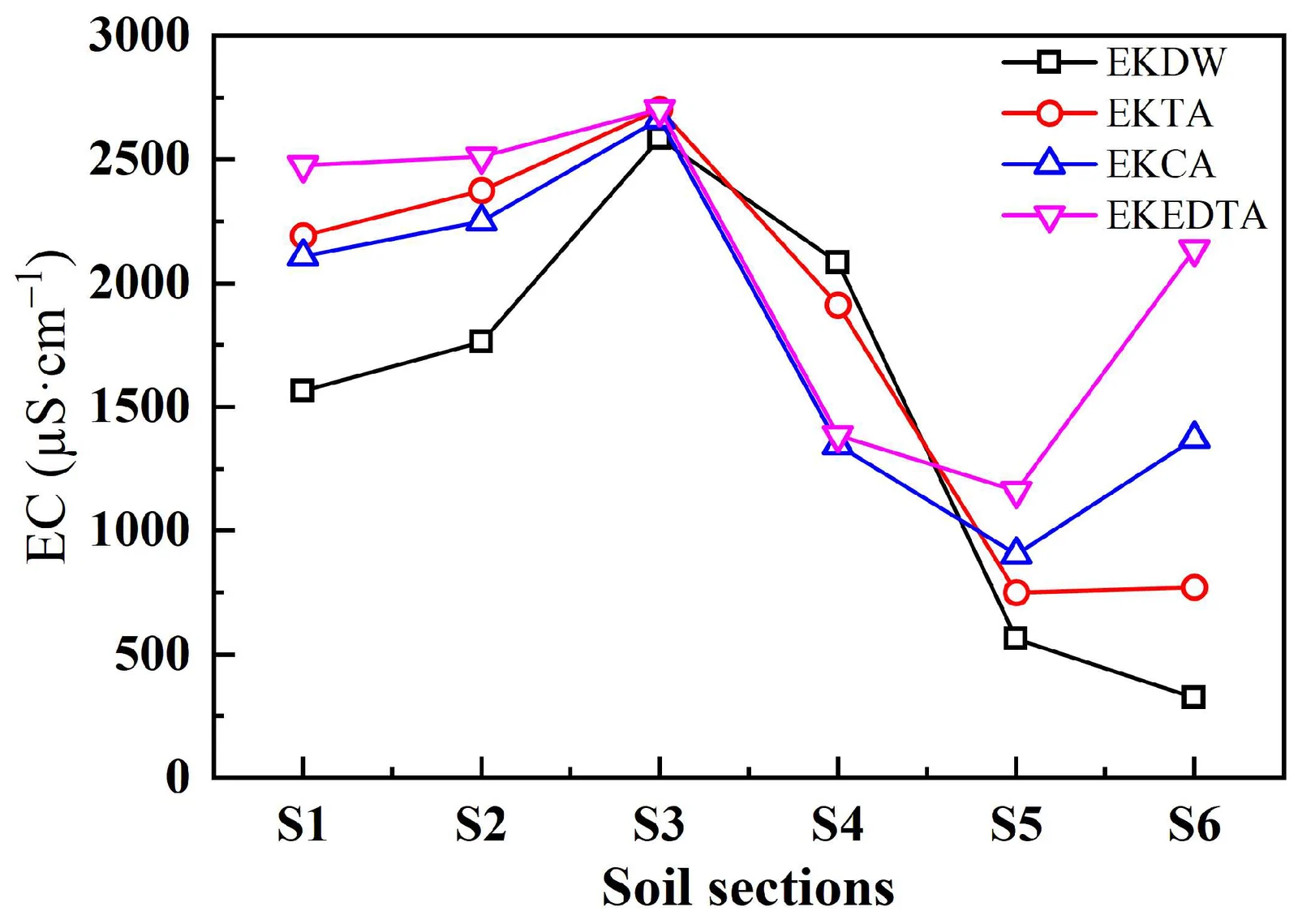
Distribution of electrical conductivity in different soil sections after electrokinetic remediation.

The EC distribution across different soil sections after EK remediation is shown in Figure 4. Overall, the EC profile of EK1 increased first and then decreased along the sample, whereas those of the EKTA, EKCA, and EKEDTA treatments showed a pattern of initial increase, subsequent decrease, and final increase. Soil EC mainly reflects the amount of mobile ions present in the soil, and the differences in EC distribution among sections were primarily associated with ion desorption, migration, and speciation transformation during EK remediation. For EKDW, H^+^ generated by anodic electrolysis created an acidic environment near the anode, which promoted the desorption of a large amount of ions in section S1. Under the electric field, these mobile ions continuously migrated toward the cathode, leading to a gradual increase in EC from S1 to S3. However, as the pH increased in the cathode-side section, some mobile ions, such as Cu^2+^, Pb^2+^, Ca^2+^, and Mg^2+^, reacted with OH^−^ to form insoluble precipitates, resulting in their transformation from mobile to immobile forms. In addition, the precipitation of heavy metals near the cathode may block part of the soil pores and hinder further directional ion migration. As a result, the EC gradually decreased from S4 to S6. Compared with EKDW, EKTA, EKCA, and EKEDTA treatments showed higher current intensities during the experiments, resulting in greater H^+^ production on the anode side and more pronounced ion desorption in the anodic section. In addition, TA, CA, and EDTA could all form complexes with heavy metal ions in the soil, producing negatively charged metal complexes that migrated toward the anode under the electric field. Therefore, the EC values in the anode-side section (S1–S3) of EKTA, EKCA, and EKEDTA treatments were not only higher than those in the cathode-side section, but also clearly higher than the corresponding values in EK1.

On the other hand, compared with EKDW, the use of TA, CA, and EDTA as catholytes promoted the desorption of heavy metal ions near the cathode, resulting in higher EC values in this section. Among the three chelating agents, EDTA exhibited a much stronger complexation capacity than TA and CA, and thus showed a greater ability to activate and desorb heavy metal ions. Consequently, EKEDTA exhibited the highest overall EC among the four test groups. In addition, cations such as Na^+^ and K^+^ in the soil migrated directionally toward the cathode under the electric field, which was also an important reason for the increase in EC in section S6 of EKTA, EKCA, and EKEDTA treatments. For EKEDTA, EDTA was used as the catholyte, introducing additional Na^+^ into the system and further increasing the ion concentration near the cathode. Therefore, the EC in section S6 of EKEDTA was higher than that of EKTA and EKCA. By comparison, the amounts of Na^+^ and K^+^ migrating toward the cathode in EKCA were higher than those in EKTA, and thus the EC in section S6 of EKCA was slightly higher than that in EKTA.

### 3.3. Variation in Electric Current

**Figure 5:**
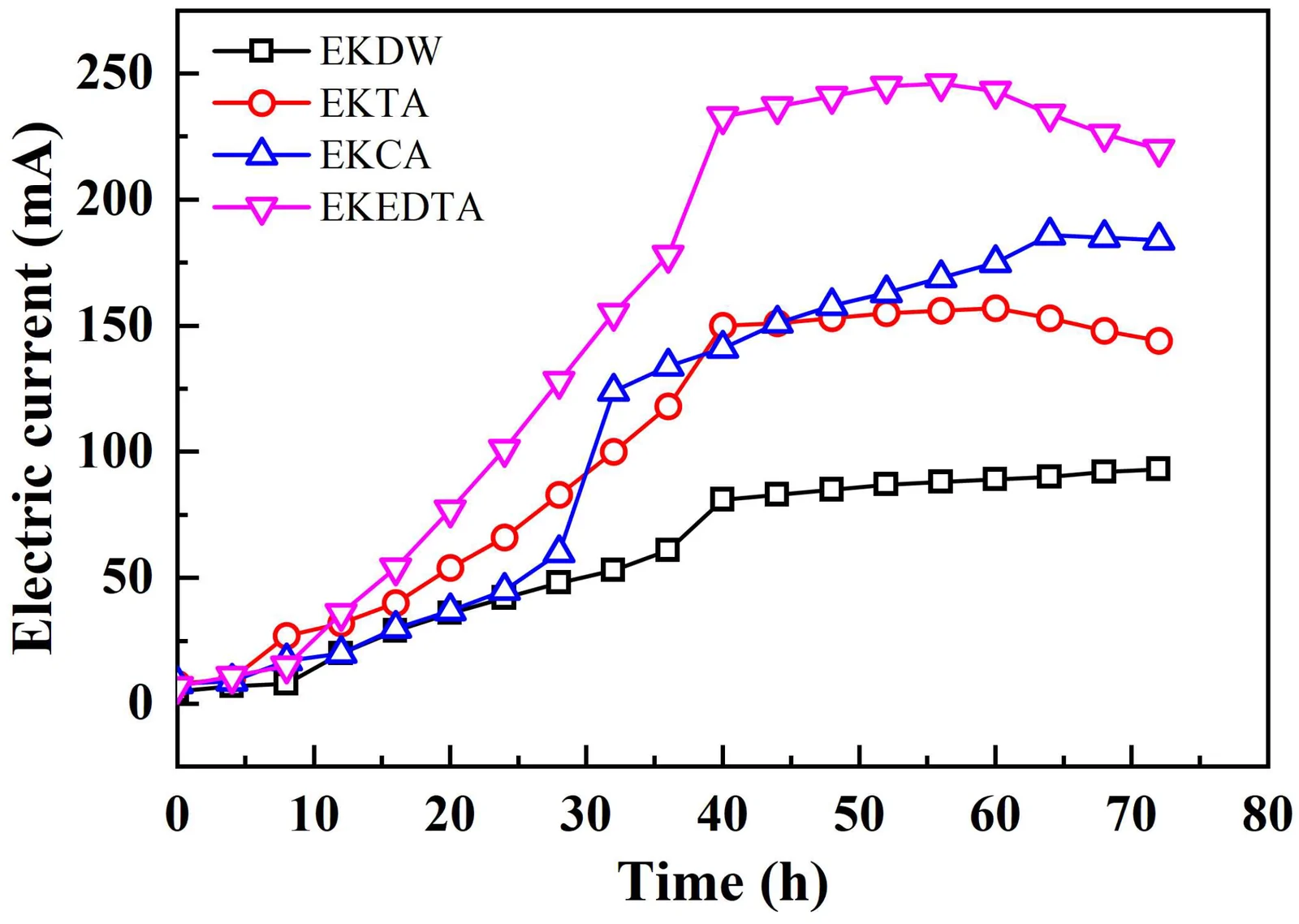
Variation in electric current during electrokinetic remediation.

Figure 5 shows the variation in electric current with time during EK remediation. Overall, the current in all four tests exhibited a similar trend, namely a gradual increase at the initial stage, followed by a relatively stable period, and then a slight decline at the later stage. Fundamentally, the current was governed by the electrical conductivity of the system, which mainly depended on the amount and mobility of the mobile ionic species in the soil. Therefore, the generation, migration, outward transport, and transformation of mobile ions into immobile forms jointly determined the evolution of soil electrical conductivity and, consequently, the variation in current.

Both H^+^ and chelating agents can promote the desorption of heavy metals from the soil, converting them from relatively stable forms into mobile ions or complexed ionic species, thereby enhancing the EC of the system. However, for loess with a strong acid-buffering capacity, the enhancement induced solely by H^+^-driven acidification was relatively limited, whereas the chelating agents played a more significant role in promoting heavy metal activation and migration. For EKDW, during the initial stage of the experiment, the continuous entry of H^+^ generated at the anode into the soil promoted the desorption of more ions from the particle surfaces into the pore solution. As a result, the amount of mobile ions in the sample gradually increased, leading to an increase in electrical conductivity and, consequently, a progressive rise in current. In the later stage, although H^+^ continued to enter the soil and further promoted ion desorption, some cations migrating toward the cathode, such as Cu^2+^, Pb^2+^, Ca^2+^, and Mg^2+^, reacted with OH^−^ generated in the cathodic section to form precipitates, causing part of the mobile ions to be converted into immobile forms. At this stage, a dynamic balance was gradually established between ion release and precipitation loss, and the total amount of mobile ions in the system tended to stabilize. Accordingly, the current also became relatively stable. No obvious current decay was observed in EKDW. On the one hand, the anode-side section (S1–S3) still maintained relatively higher EC, so the total resistance of the series system did not increase markedly. On the other hand, because the experimental duration was relatively short, the precipitates formed near the cathode had not yet completely blocked the soil pores, and ion migration pathways still remained within the soil.

For the EKTA, EKCA, and EKEDTA treatments, the addition of chelating agents significantly enhanced the desorption and migration of heavy metals through complexation, thereby markedly increasing the amount of mobile ions in the system. As a result, the overall current in these three groups was higher than that in EKDW. Among them, EDTA exhibited a much stronger chelating ability than TA and CA, enabling EKEDTA to activate more heavy metal ions and thus producing the highest current. By contrast, the chelating capacities of CA and TA were similar, and therefore the difference in current between EKCA and EKTA was relatively small. In the later stage of the experiments, as a large number of ions gradually migrated out of the soil sample into the electrode electrolytes, the total amount of mobile ions remaining in the soil decreased, leading to a slight reduction in overall soil EC. Consequently, the currents in EKTA, EKCA, and EKEDTA treatments all showed a slight decline.

### 3.4. Variation in EOF

Under a direct-current electric field, cations in the diffuse double layer surrounding clay particles migrate toward the cathode and, through hydration, drag associated water molecules in the same direction, thereby generating electroosmotic flow [30]. This process is generally manifested as a forward electroosmotic flow from the anode to the cathode. However, when the soil is under a low-pH environment, its Zeta potential may shift from negative to positive, which can reverse the direction of electroosmotic flow and produce a backward flow from the cathode to the anode [33,34].

**Figure 6:**
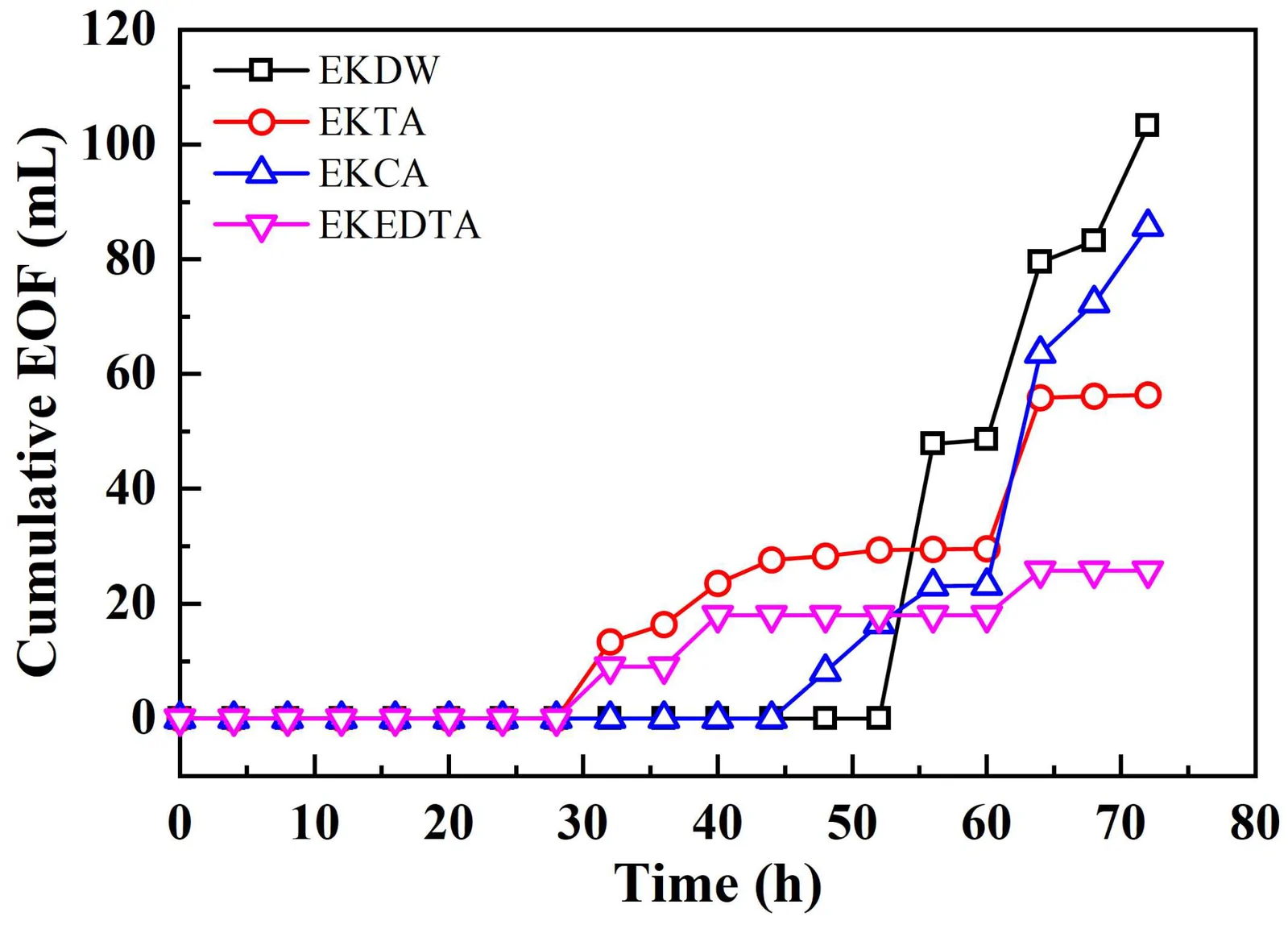
Relationships of accumulated electroosmosis flow (EOF) versus time under the effect of different chelating agents.

Figure 6 shows the variation in cumulative EOF with time during EK remediation. Overall, the cumulative EOF in all four tests increased progressively with time, although the rate of increase gradually decreased. The magnitude of cumulative EOF was jointly influenced by current intensity, soil pH, and the migration behavior of hydrated ions. In general, a higher current, a higher soil pH, and a greater amount of hydrated cations migrating toward the cathode favored forward EOF. In contrast, lower pH and a larger amount of hydrated anions migrating toward the anode tended to weaken the forward electroosmotic flow. At the initial stage of the experiments, no obvious EOF was observed in any of the four groups because of the strong adsorption of water molecules by the soil and the relatively low current intensity. Comparatively, EKTA and EKEDTA exhibited higher current in the early stage, whereas EKDW and EKCA showed lower current. As a result, electroosmotic flow appeared earlier in EKTA and EKEDTA and later in EKDW and EKCA. In the middle and late stages of the experiments, particularly after 28 h, the cumulative EOF in all the groups increased markedly as the current gradually rose. Yue Song et al. [30] reported that an increase in soil pH enlarges the absolute value of the Zeta potential, that is, the surface potential becomes more negative, while the diffuse double layer surrounding clay particles becomes thicker, both of which are favorable for EOF. Because EKDW maintained a relatively high overall soil pH, it showed the highest cumulative EOF among the four groups. Although the currents in EKTA, EKCA, and EKEDTA treatments were all higher than that in EKDW, their cumulative EOF did not increase accordingly, indicating that under chelator-amended conditions, current was not the only factor controlling EOF. For the EKTA, EKCA, and EKEDTA treatments, heavy metal ions complexed with TA, CA, and EDTA mainly existed as negatively charged metal complexes and migrated toward the anode, rather than moving as cations toward the cathode. This weakened the forward electroosmotic flow. In addition, all three chelating agents contain hydrophilic functional groups such as carboxyl groups. Their anionic forms can bind water molecules to form hydrated anions, which then migrate toward the anode, thereby partially offsetting water migration toward the cathode [28]. Therefore, despite the higher current observed in the EKTA, EKCA, and EKEDTA treatments, their cumulative EOF were all lower than that of EKDW. A further comparison shows that EDTA had a much stronger complexation capacity than TA and CA. Therefore, in EKEDTA, a larger amount of metal complexes migrating toward the anode was formed. Meanwhile, EDTA contains more carboxyl groups and can bind more water molecules, resulting in a more pronounced weakening effect on forward EOF. Consequently, EKEDTA exhibited the lowest cumulative EOF among the four groups. By comparison, the current in EKCA was slightly higher than that in EKTA during the later stage of the experiment, and thus its cumulative EOF was also slightly higher than that of EKTA.

### 3.5. Removal Efficiency

**Figure 7:**
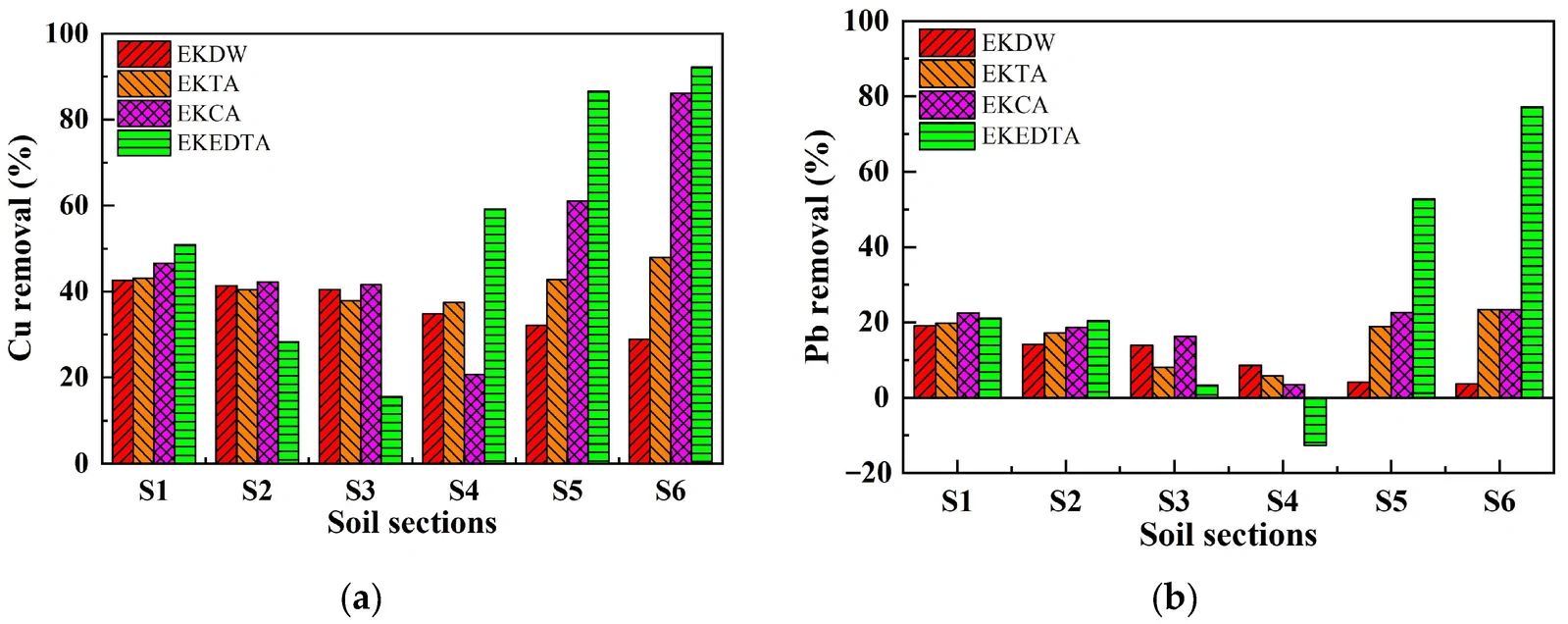
Removal of heavy metals across six soil sections after the EK remediation: (**a**) copper removal and (**b**) lead removal.

Figure 7 illustrates the removal efficiencies of Cu and Pb in different sections during EK remediation. Overall, Cu was removed more effectively than Pb from the loess samples. This difference can be mainly attributed to the stronger adsorption and fixation of Pb by loess, which makes its desorption and migration under an electric field more difficult [30]. For EKDW, the removal efficiency of Cu gradually decreased from 42.6% in S1 to 29.3% in S6, while that of Pb decreased from 18.9% to 3.7%, both showing a progressive decline from the anode side to the cathode side. This was mainly attributed to the fact that, during the later stage of the experiment, some heavy metal ions migrating toward the cathode precipitated with OH^−^ under high-pH conditions and accumulated in the pores near the cathode, thereby blocking the pore channels and hindering the further migration of heavy metals [35]. As a result, the removal performance of both Cu and Pb gradually deteriorated with decreasing distance to the cathode. In the chelator-amended groups (EKTA, EKCA, and EKEDTA treatments), the removal efficiencies of Cu and Pb in different sections were generally higher near the anode and cathode, but lower in the middle sections. Near the cathode, heavy metals were desorbed from the soil particle surfaces through complexation with TA, CA, and EDTA, and mainly migrated toward the anode as negatively charged metal complexes. In contrast, near the anode, heavy metals were primarily desorbed under H^+^-induced acidification and migrated toward the cathode in cationic form. Therefore, the sections adjacent to the electrodes provided favorable conditions for heavy metal activation and migration and thus exhibited relatively high removal efficiencies, whereas the middle sections were more strongly constrained by ion convergence and mass transfer limitations, leading to lower removal efficiencies. Further comparison revealed that section S6 exhibited the highest removal efficiencies for both Cu and Pb in EKTA, EKCA, and EKEDTA treatments, indicating that, in loess with a strong acid-buffering capacity, chelating agents near the cathode exerted stronger activation and desorption effects than H^+^-induced desorption near the anode. This suggests that chelator-enhanced treatment is an effective approach to alleviating the restricted heavy metal migration in the cathodic section of loess. Among the three chelating agents, EDTA showed the most pronounced enhancement effect, particularly in improving the removal of Cu and Pb near the cathode. Although EKEDTA treatment showed some local accumulation, with Cu enriched around section S3 and Pb around sections S3 and S4, it still achieved the best overall remediation performance among the four groups. The total removal efficiencies reached 55.4% for Cu and 27.2% for Pb. This was mainly attributed to the multidentate structure of EDTA, which enables the formation of highly stable complexes with heavy metal ions, thereby markedly enhancing both their desorption from loess and their migration under the electric field. Consequently, EKEDTA exhibited the greatest overall enhancement in remediation performance.

### 3.6. Discussion

**Figure 8:**
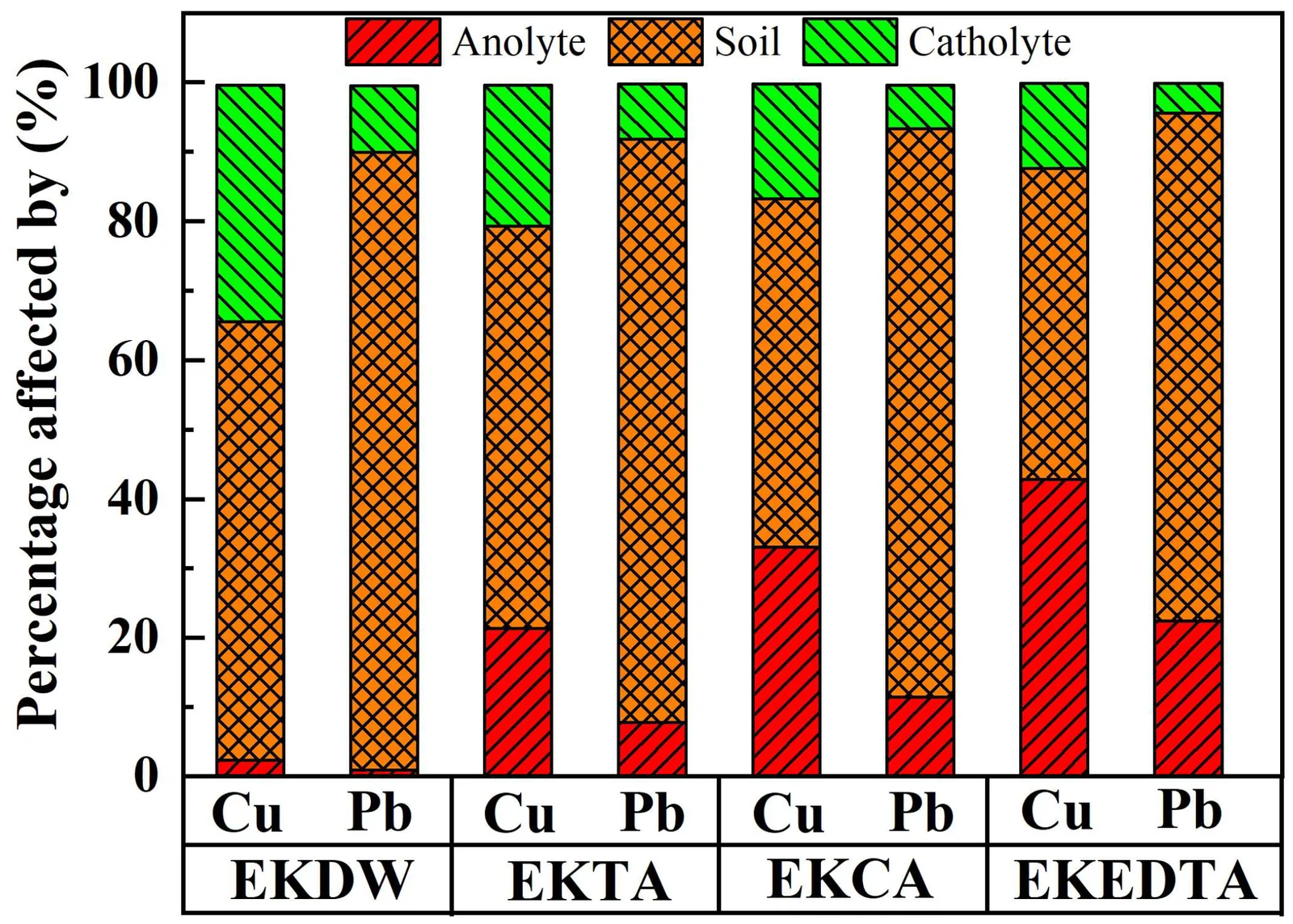
Mass balance of Cu and Pb after EK remediation.

A combined analysis of the removal efficiencies and metal mass balance shows that chelators altered the direction and extent of Cu and Pb transport under the electric field [32,33,34]. Figure 8 presents the distributions of Cu and Pb remaining in the soil and recovered in the anolyte and catholyte after treatment. In the DW control, metal desorption was limited, and most Cu and Pb remained in the soil. The small mobile fraction mainly migrated toward the cathode as cations, resulting in greater metal recovery in the catholyte than in the anolyte [35,36,37,38].

In the EKTA, EKCA, and EKEDTA treatments, the addition of chelating agents promoted the desorption of Cu and Pb from the soil. As a result, the residual heavy metal contents in the samples decreased to different extents. After complexation with the chelating agents, heavy metal ions formed negatively charged complexes. These complexes mainly migrated toward the anode under the electric field. Consequently, the heavy metal concentrations in the anolyte increased, whereas those in the catholyte decreased. Among the three chelating agents, EKEDTA exhibited the strongest complexation capacity and the greatest ability to promote heavy metal desorption from the soil. In addition, the complexes formed between EDTA and Cu or Pb were more stable and carried stronger negative charges. This gave them a greater tendency to migrate toward the anode during EK remediation. Therefore, the EKEDTA treatment showed the lowest residual heavy metal contents in the sample and in the catholyte, but the highest heavy metal concentrations in the anolyte. This indicates that EDTA provided the most significant enhancement in the EK remediation of Cu- and Pb-contaminated loess.

**Figure 9:**
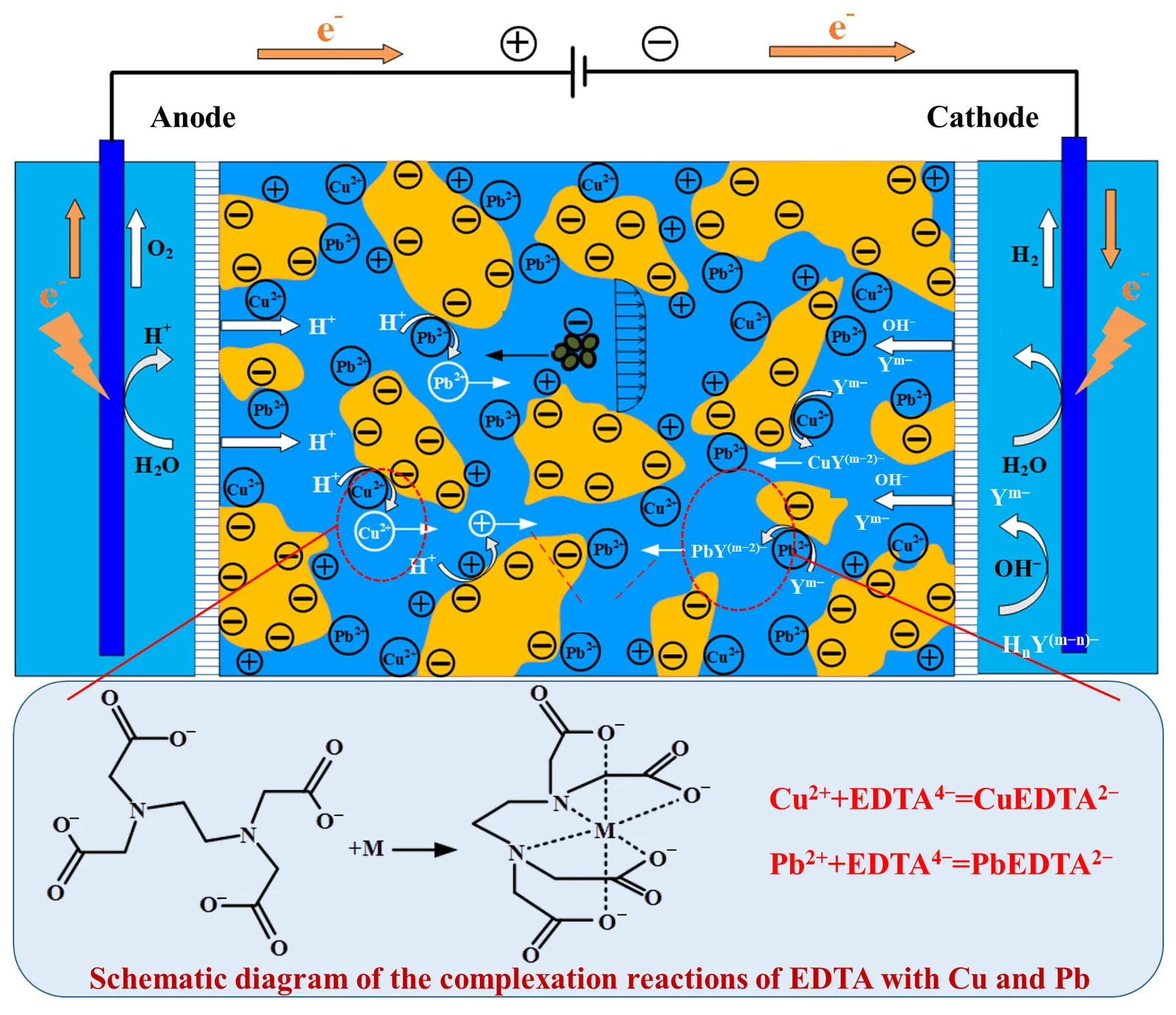
Schematic diagram of EDTA-enhanced electrokinetic remediation.

The experimental results showed that under TA- and CA-enhanced conditions, the overall removal efficiencies of Cu were 41.9% and 49.8%, respectively, whereas those of Pb were 15.7% and 18.0%, respectively. In contrast, under EDTA-enhanced conditions, the overall removal efficiencies of Cu and Pb reached 55.4% and 27.2%, respectively. These results clearly indicate that EDTA was markedly more effective than tartaric acid and citric acid in enhancing the electrokinetic remediation of Cu- and Pb-contaminated loess. Therefore, the enhancement mechanism of EDTA was further analyzed, as schematically illustrated in Figure 9. In Figure 9, Y represents the EDTA molecule, and m denotes the maximum negative valence that EDTA can attain after dissociation. For EDTA, m = 4. n represents the number of H^+^ ions associated with the EDTA molecule, and for EDTA, n can be 0, 1, 2, 3, or 4.

When EDTA was used as the catholyte, the weakly acidic EDTA first reacted with the OH^−^ generated by cathodic electrolysis and gradually formed negatively charged EDTA anions, mainly in the form of EDTA^4−^. Under the electric field, these anions migrated toward the anode. After entering the soil, they preferentially complexed with the heavy metal ions that had already been desorbed or could be activated in the cathode-side section, thereby forming negatively charged metal-EDTA complexes. Because these complexes possessed strong electromigration ability, they continued to migrate toward the anode and eventually entered the anolyte, resulting in the transfer of heavy metals from the soil into the external electrolyte system. Meanwhile, part of the EDTA anions that had not yet participated in complexation continued to migrate toward the anode-side section. Before their arrival, the H^+^ generated by anodic electrolysis had already promoted the desorption of heavy metals near the anode, converting Cu^2+^ and Pb^2+^ originally adsorbed on soil particle surfaces into free metal cations. These metal cations then migrated toward the cathode under the combined effects of electromigration and forward electroosmotic flow. During migration, they further complexed with EDTA anions, which altered both their migration form and migration direction. In Figure 9, M represents Cu^2+^ or Pb^2+^.

From a molecular perspective, EDTA contains four carboxyl oxygen atoms and two amino nitrogen atoms, enabling it to form multidentate coordination structures with metal ions and generate highly stable metal complexes with multiple chelate rings [39,40]. These complexes remain stable over a wide pH range and are less likely to precipitate or be readsorbed. As a result, EDTA can significantly enhance both the activation of heavy metals in soil and their migration under the electric field. Owing to its combined abilities to promote desorption, stabilize metal complexes, and enhance migration, EDTA showed the most pronounced improvement in the removal of Cu and Pb. Overall, the enhancement of electrokinetic remediation by EDTA can be attributed to a synergistic mechanism involving complexation-induced activation in the cathode section, directional migration of metal-EDTA complexes, and acidification-assisted desorption in the anode section. Through this combined effect, EDTA effectively alleviated the inherent limitations of heavy metals in loess, namely difficult desorption and limited migration, thereby significantly improving the overall electrokinetic remediation efficiency.

### 3.7. Practical Implications and Limitations

The laboratory results indicate that chelator-enhanced EK treatment is especially suitable for low-permeability loess, where excavation causes major disturbance and conventional hydraulic flushing is inefficient. Table 3 qualitatively compares the principal alternatives. EK treatment offers controllable in situ transport and a shorter treatment period than phytoremediation, but it requires power, electrodes, electrolyte circulation, pH control, and management of extracted solutions. Chemical leaching may be faster, but it can consume large reagent volumes and alter soil properties, whereas excavation transfers contaminated soil to another treatment or disposal location.

In practice, EDTA is preferred when maximum Cu and Pb removal is the primary objective, whereas TA or CA may be preferable when biodegradability and residual-chelator risk are prioritized. The tested dosage of 0.1 mol/L should not be treated as universally optimal; field dosage should be selected through bench-scale treatability tests considering carbonate content, pH-buffering capacity, metal concentration, and electrolyte recovery. Recommended monitoring includes current, voltage, pH, electrical conductivity, EOF, metal concentrations in the anolyte and catholyte, residual total and leachable metals, and post-treatment hydraulic and mechanical properties. Long-term studies should evaluate chelator persistence, possible remobilization of metal-chelate complexes, pore-structure changes, and the stability of remediation over months to years.

## 4. Conclusions

This study investigated the effects of three chelating agents on ion migration during the electrokinetic remediation of Cu- and Pb-contaminated loess. Combined with the results for pH, electrical conductivity, current, electroosmotic flow, removal efficiency, and heavy metal speciation, the following conclusions can be drawn: Based on the results and discussion, the following main conclusions can be drawn:

(1) During the EK remediation of Cu- and Pb-contaminated loess, the overall removal efficiency of Cu was higher than that of Pb. This was mainly because loess has a stronger adsorption and fixation capacity for Pb than for Cu, which makes Pb more difficult to desorb and migrate in the soil and thus limits its EK removal efficiency.

(2) TA, CA, and EDTA all promoted the desorption and activation of Cu and Pb in loess through complexation and formed charged metal complexes, thereby enhancing heavy metal migration in the soil. As a result, soil electrical conductivity and system current increased, leading to improvements in the EK removal of Cu and Pb to different extents. This enhancement was particularly evident in the cathode-side section.

(3) EDTA produced the greatest enhancement, despite some local metal accumulation in the middle sections. The overall removal efficiencies reached 55.4% for Cu and 27.2% for Pb. Its strong multidentate complexation stabilized negatively charged metal-EDTA complexes, promoted transport toward the anode, and increased metal recovery in the anolyte. Field application should additionally control energy use, residual chelator, extracted electrolyte, and long-term remobilization risk.

## Figures and Tables

**Table 2:** Experimental design of the enhanced electrokinetic remediation of copper and lead-contaminated loess.

Test	Electrode Type	Pollutant	Voltage Gradient/Vcm^−1^	Anolyte	Catholyte	Duration Time/h
EKDW	EKG 0.125	Cu + Pb	1.5	DW	DW	72
EKTA	EKG 0.125	Cu + Pb	1.5	DW	TA	72
EKCA	EKG 0.75	Cu + Pb	1.5	DW	CA	72
EKEDTA	EKG 0.75	Cu + Pb	1.5	DW	EDTA	72

Note: DW indicates deionized water; TA indicates tartaric acid; CA indicates citric acid; and EDTA indicates disodium ethylenediaminetetraacetate.

**Table 3:** Practical comparison of remediation options for Cu- and Pb-contaminated loess.

Method	Typical Time Frame	Suitability for Loess	Main Advantages	Main Limitations
Excavation/replacement	Immediate to weeks	Applicable but highly disruptive	Rapid risk removal; simple verification	High transport and disposal cost; land disturbance; contaminant transfer
Chemical leaching	Days to weeks	Limited by low permeability and buffering	Rapid metal solubilization	High reagent/water demand; secondary wastewater; possible soil-property damage
Phytoremediation	Months to years	Limited by rooting depth and climate	Low energy use; low disturbance	Slow; seasonal; weak for strongly bound Pb
Chelator-enhanced EK	Days to weeks	Well suited to low-permeability loess	In situ control; directional transport; limited excavation	Power and hardware needs; pH control; residual chelator and metal-complex management

## Data Availability

The original contributions presented in this study are included in the article. Further inquiries can be directed to the corresponding authors.
